# Genome-wide DNA methylation analysis in lung fibroblasts co-cultured with silica-exposed alveolar macrophages

**DOI:** 10.1186/s12931-017-0576-z

**Published:** 2017-05-12

**Authors:** Juan Li, Wu Yao, Lin Zhang, Lei Bao, Huiting Chen, Di Wang, Zhongzheng Yue, Yiping Li, Miao Zhang, Changfu Hao

**Affiliations:** College of Public Health, Zhengzhou University, No.100, Kexue Road, Zhengzhou city, Henan province China

**Keywords:** Fibroblast, Crystalline silica, Methylation, Differentiation

## Abstract

**Background:**

Exposure to crystalline silica is considered to increase the risk of lung fibrosis. The primary effector cell, the myofibroblast, plays an important role in the deposition of extracellular matrix (ECM). DNA methylation change is considered to have a potential effect on myofibroblast differentiation. Therefore, the present study was designed to investigate the genome-wide DNA methylation profiles of lung fibroblasts co-cultured with alveolar macrophages exposed to crystalline silica in vitro.

**Methods:**

AM/fibroblast co-culture system was established. CCK8 was used to assess the toxicity of AMs. mRNA and protein expression of collagen I, α-SMA, MAPK9 and TGF-β1 of fibroblasts after AMs exposed to 100 μg /ml SiO_2_ for 0–, 24–, or 48 h were determined by means of quantitative real-time PCR, immunoblotting and immunohistochemistry. Genomic DNA of fibroblasts was isolated using MeDIP-Seq to sequence. R software, GO, KEGG and Cytoscape were used to analyze the data.

**Results:**

SiO_2_ exposure increased the expression of collagen I and α-SMA in fibroblasts in co-culture system. Analysis of fibroblast methylome identified extensive methylation changes involved in several signaling pathways, such as the MAPK signaling pathway and metabolic pathways. Several candidates, including *Tgfb1 and Mapk9*, are hubs who can connect the gene clusters. MAPK9 mRNA expression was significantly higher in fibroblast exposed to SiO_2_ in co-culture system for 48 h. MAPK9 protein expression was increased at both 24-h and 48-h treatment groups. TGF-β1 mRNA expression of fibroblast has a time-dependent manner, but we didn’t observe the TGF-β1 protein expression.

**Conclusion:**

*Tgfb1* and *Mapk9* are helpful to explore the mechanism of myofibroblast differentiation. The genome-wide DNA methylation profiles of fibroblasts in this experimental silicosis model will be useful for future studies on epigenetic gene regulation during myofibroblast differentiation.

**Electronic supplementary material:**

The online version of this article (doi:10.1186/s12931-017-0576-z) contains supplementary material, which is available to authorized users.

## Background

Chronic inhalation of crystalline silica is associated with the development of silicosis, which is characterized by inflammation and lung fibrosis [1, 2]. A multitude of cell types are well known to be involved in the process of pulmonary fibrosis. The critical cell involved in lung fibrosis is the myofibroblast, which is recruited to and accumulates at the injured site [3]. The lung resident fibroblast is widely regarded as the major source of myofibroblast. Transdifferentiation is defined as one differentiated cell losing its surface phenotypes and switching to another type of normal differentiated cell [4]. Lung local fibroblasts have been shown to transform under the influence of cytokines, such as transforming growth factor-β1 (TGF-β1), into myofibroblasts with the ability to overrepresent and to secrete excessive ECM [5, 6]. Although much is known about the mechanistic aspects of fibroblast differentiation into myofibroblasts, the underlying mechanisms remain unclear [7, 8].

DNA methylation, a major epigenetic mechanism, is one of the most extensively studied epigenetic mechanisms that regulate gene expression. Accumulating evidence suggests that epigenetic DNA alterations play a crucial role in differentiation [9–11]. However, explorations into its potential role in lung fibroblasts co-cultured with silica-exposed alveolar macrophages switching to a myofibroblast have been limited. Several genes, such as *MeCP2*, have been shown to be differentially methylated during the myofibroblast differentiation of a hepatic stellate cell [12], but a global analysis of methylation differences in silica-exposed myofibroblast differentiation in the co-culture model has not been reported.

In the present study, we used Methylated DNA Immunoprecipitation (MeDIP) coupled with next-generation sequencing to profile whole-genome DNA methylation patterns of fibroblasts from an in vitro co-culture model. We analyzed the data to identify possible biological pathways that allow fibroblasts to differentiate into myofibroblasts as well as the interactions of related genes.

## Methods

### Animals

The animal experiments were approved by the Experimental Animal Ethics Committee of Zhengzhou University, and our study was conducted in accordance with the guidelines of the Chinese Association of Laboratory Animal Care. Experiments were performed using male Sprague–Dawley (SD) rats (age, 6–8 weeks; weight, 180–220 g). The rats were purchased from the animal center of Henan province (SCXK 2015–0004, Henan, China). The rats were housed under controlled temperature (22 ± 2 °C) and exposed to a 12 h light–dark cycle. All rats were provided with free access to standard rat feed and tap water.

### Cell culture

Lung fibroblasts were isolated from SD rats as previously described [13]. Briefly, rats were anesthetized with chloral hydrate and then sacrificed by cervical dislocation to remove the lung. The lung was washed three times with D-Hank’s solution, minced into small pieces and digested with 0.25% trypsin. Lung fibroblasts were cultured in Dulbecco’s Modification of Eagle’s medium Dulbecco (DMEM) supplemented with 10% heat-inactivated fetal bovine serum (HyeClone, USA) at 37 °C and 5% CO_2_. After 24 h, the medium was replaced and subsequently refreshed twice per week.

After chloral hydrate anesthesia and cervical dislocation, the rat macrophages (AMs) were collected from the SD rats by bronchoalveolar lavage (BAL). Tracheas were cannulated, and BAL was performed with 5 ml of cold sterile D-Hank’s solution. After the cell numbers were counted, the cells were incubated at 37 °C for 2 h at 5% CO_2_, and fresh medium was added. Rat AM/fibroblast co-culture system was established (See Additional file 1): a co-culture system was carried out using permeable membrane inserts in 6-well Transwell microplate supports (0.4 μm pore size polyester membrane precoated with poly-L-lysine; Corning, NY, USA). Fibroblasts were seeded into six-well plates and incubated for one or two days until approximately 60% confluence was achieved. The fibroblasts did not contact with the SiO_2._ AMs were exposed to 100 μg/ml SiO_2_ (median diameter, 1–5 μm; Sigma Aldrich, St. Louis, USA) in the insert which placed into the six-well dish for 0-, 24-, or 48 h after the overnight culture. The fibroblasts were prepared for immunohistochemical staining and genomic DNA distracted.

### Cytotoxicity analysis

CCK8 (Dojindo, Kumamoto, Japan) was used to measure the cytotoxicity of SiO_2_ on AMs. The AMs were seeded in 96-well plate (5 × 10^4^ cells/ well) and then treated with concentrations of SiO_2_ (0, 20, 40 60, 80, 100, 120 and 140 μg/ml) for 24- and 48 h, respectively. 10 μl CCK8 was added into each well and incubated at 37 °C for 1 h. Then, microplate reader (Tecan Infinite M200, Tecan, Wetzlar, Germany) was used to determine the absorbance at 450 nm according to the manufacturer’s instructions.

### RNA isolation and qPCR

After AMs exposed to 100 μg/ml SiO_2_ in co-culture system for 0–, 24–and 48 h, total RNA of fibroblasts was extracted using Trizol (TaKaRa, Tokyo, Japan) RNA Isolation Protocol. The first-strand cDNA was synthesized using PrimeScriptTM RT reagent kit (TaKaRa, Tokyo, Japan) according to the manufacturer’s protocols. Collagen I, α-SMA, MAPK9 (JNK2) and TGF-β1 mRNA expression was assessed by Mx3000P QPCR System (Stratagene, California, USA) with the SYBR Premix ExTM Taq (Tli RNaseH Plus) kit (TaKaRa, Tokyo, Japan) and gene specific primers (Sangon Biotech, Shanghai, China) as indicated in Table 1 . All values were normalized to the transcription of the housekeeping gene glyceraldehyde-3-phosphate dehydrogenase (GAPDH). Mean fold changes in mRNA expression were calculated by the 2^-ΔΔCT^ method.

**Table 1:** Primers for qPCR

Gene symbol	Accession number	Sequence [5′ to 3′]	Amplicon length [bp]
Col1a1	NM_053304.1	for-CGTGGAAACCTGATGTATGCT	169
		rev-CCTATGACTTCTGCGTCTGG	
Acta2	NM_031004.2	for-CTTCAATGTCCCTGCCATGT	103
		rev-AGTCACGCCATCTCCAGAGT	
Tgfb1	NM_021578.2	for-ATGACATGAACCGACCCTTC	121
		rev-TTCTCTGTGGAGCTGAAGCA	
Mapk9	NM_001270545.1	for-ATGATGCCCAGTTGGAAGAA	120
		rev-GTGCTGAAGGCTGGTCTTTC	
Gapdh	NM_017008.4	for-GGCACAGTCAAGGCTGAGAATG	143
		rev-ATGGTGGTGAAGACGCCAGTA	

### Immunoblotting

The fibroblasts were lysed with protein lysis buffer ((Beijing dingguo changsheng biotech CO.LTD, Beijing, China)). Protein concentration was measured using BCA Protein Assay Kit (Beijing dingguo changsheng biotech CO.LTD, Beijing, China). Protein samples were mixed with 4 × loading buffer and boiled for 5 min at 95 °C. Proteins were separated by 8% SDS-PAGE and transferred to polyvinylidene fluoride membrane. Membranes were blocked by 5% nonfat dry milk in 1× TBS, 0.1% Tween-20 for 1 h at room temperature, then incubated at 4 °C overnight with anti-Collagen I polyclonal Ab (1:2000 dilution, Abcam), or anti-α-SMA (1:2000 dilution, Abcam) or anti-GAPDH mAb. After that, the membranes were incubated with corresponding secondary antibodies for 1 h at room temperature. ECL enhanced Chemiluminescence Kit (Beyotime Biotechnology, Shanghai, China) plus Amersham Imager 600 western blotting detection system (Amersham, Uppsala, Sweden) was used to detect the bands. Signals were quantified using the Image J software.

### Immunohistochemistry (IHC)

The fibroblasts grown on cover slips were fixed in 4% paraformaldehyde (Sigma, St. Louis, MO, USA) and permeabilized with 0.3% triton X-100 in PBS. After blocking the non-specific binding by 10% goat serum for 30 min at room temperature, the coverslips were incubated with anti-Collagen I polyclonal Ab (1:400 dilution, Rockland, PA, USA), anti-α-SMA mAb (1:200 dilution, Wuhan Boster Biological Technology, Ltd., Wuhan, China), anti-TGF-β1 polyclonal Ab (1:100 dilution, Wuhan Boster Biological Technology, Ltd., Wuhan, China) and anti-JNK2 mAb (1:100 dilution, Abcam) at 4 °C for overnight, respectively. As secondary antibody, HRP labeled goat anti-mouse IgG or HRP labeled goat anti-rabbit IgG (1:200 dilution, Wuhan Boster Biological Technology, Ltd., Wuhan, China) were used for 1 h at room temperature. The cover slips were rinsed and reacted with 3,3′-diaminobenzidine (DAB). Positive IHC staining was presented as brown staining. Image-Pro Plus 6.0 software was used to quantify (average optical density (AOD) in 5 high power vision fields, AOD = Integrated Optical Density (IOD) SUM/Area SUM).

### DNA Isolation and MeDIP-Seq

Genomic DNA of fibroblasts was isolated using the DNeasy Kit (Qiagen, CA) according to the manufacturer’s protocol. The DNA quality was evaluated using a NanoDrop spectrophotometer and an Agilent Bioanalyzer 2100. Only high-quality DNA (260/280 > 1.8) was used in this study. In total, 3 samples were used for construction of the methylated sequences using the Illumina HiSeq 2000 sequencing system. 1 μg of genomic DNA was sheared into 150–500 bp fragments using the Covaris S220 system (Covaris, MA, USA). According to the manufacturer’s protocol, the fragmented DNA was end-repaired, A-tailed, and ligated to an adapter and purified using the Agencourt AMPure XP kit. The subsequent MeDIP enrichment used 0.4 μg DNA. Briefly, immunoprecipitation was carried out at 37 °C for 0.5–1 h. After amplification and purification, we performed agarose gel electrophoresis and excised bands from the gel to produce libraries with insert sizes of ~125 bp, then quantified these libraries using the KAPA DNA Reagent and Kits (KAPA Biosystem). The total libraries randomly arranged in 8 lanes were sequenced by Next Generation Sequencing. The MeDIP-seq data can be accessed in GEO under GSE93116.

### Sequence filtering, mapping and peaks scanning

A Perl program was used to filter off low quality sequences from raw sequencing data. The quality of each base was checked from the first base of each read. Once a low-quality base (quality < 10) was found, it was removed together with following sequences. For paired-end reads, if one read was less than 30 bases after the trimming of low quality bases, the whole paired-end reads were removed. Then, the remaining high quality reads of a sample were mapped by bowtie software 0.12.8 [14] to the Rat genome downloaded from Ensembl. After the alignment of high quality reads to the reference genome, the Model-based Analysis of MeDIP-Seq software (MACS) [15] was used to scan methylated peaks. The locations of unique reads in the reference genome were summarized and then for further analysis (Addition files 2 and 3).

### Identification of differentially methylated regions (DMRs)

The normalized read counts were used to measure level of methylated peaks to allow for comparison between experiments. Transcripts Reads Number per Million (TPM) method was used to normalize the read counts. The formula is:

$$ \mathrm{T}\mathrm{P}\mathrm{M} = 1000000\times \left(\mathrm{The}\ \mathrm{Transcript}\ \mathrm{M}\mathrm{apped}\ \mathrm{Reads}\right)\ /\left(\mathrm{Total}\ \mathrm{M}\mathrm{apped}\ \mathrm{Reads}\right) $$

Then, the regions of differential methylation (DMRs) were identified by applying the edgeR software integrated in the Bioconductor DiffBind package [16]. DMRs were deemed with a *P* value<0.05, FDR<0.05 and a 2 fold change in sequence counts (Empirical Bayes estimation and exact tests based on the negative binomial distribution). The regions of differential methylation associate genes (DMGs) were obtained by annotation of hypermethylated and hypomethylated DMRs. Then, we obtained DMGs of 24 h group (compared with control group) and 48 h group (compared with control group) separately for further analysis.

### Validation of methylation status by bisulfite sequencing PCR (BSP)

To confirm the results obtained from the MeDIP-sequence, genomic DNA was treated with bisulfite using EZ DNA methylation Gold kit (Zymo Research) according the manufacturer’s instructions. Two pairs of primers were designed using Methprimer [17] in order to obtain pure products by PCR (See Additional file 4 for primer sequences and information of PCR). Then PCR products were cloned into the pUCm-T Vector. After plasmid reproduced and bacterial culture, the positive clones were used to sequence.

### Functional annotation and pathway enrichment analysis

WEGO (Web Gene Ontology Annotation Plot) [18] and WebGestalt (http://www.webgestalt.org/) were applied for Gene ontology and Pathway Enrichment analysis, with *P*<0.05 and Benjiamini adjusted *P*<0.05. PANTHER website (http://go.pantherdb.org/) and Kyoto Encyclopedia of Genes and Genomes (KEGG) (http://www.kegg.jp/) were also used to classify gene function and gene products.

### Integration of protein-protein interaction (PPI) network and module analysis

Cytoscape 3.2.0 [19] was used to evaluate the interactive relationships among DEGs, A PPI network was built by plugin string. The experimentally validated interactions with a combined score>0.4 were selected as significant. The plug-in Molecular Complex Detection (MCODE) was used to screen the modules of PPI network in Cytoscape. The criteria were set as follows: MCODE scores > 3 and number of nodes> 4. In addition, the function and pathway enrichment analysis were performed for DEGs in module 2. *P*<0.05 was considered to have significant difference.

### Statistical analysis

Data are presented as means ± SD. Comparisons between multiple independent groups were performed with one-way ANOVA, followed by a post hoc analysis with Bonferroni test using SPSS17.0 software. Group differences with *P* < 0.05 indicate a statistically significant difference.

## Results

### Cytotoxicity of Silica

CCK8 assay was used to evaluate the cytotoxicity of AMs exposure to SiO_2_. The viability of AMs exposed to SiO_2_ was significantly decreased by a dose-dependent manner (*P*≤0.05) (Additional file 5).

### Effect of SiO_2_ on collagen I and α-SMA mRNA and protein expression in fibroblasts

**Fig. 1:**
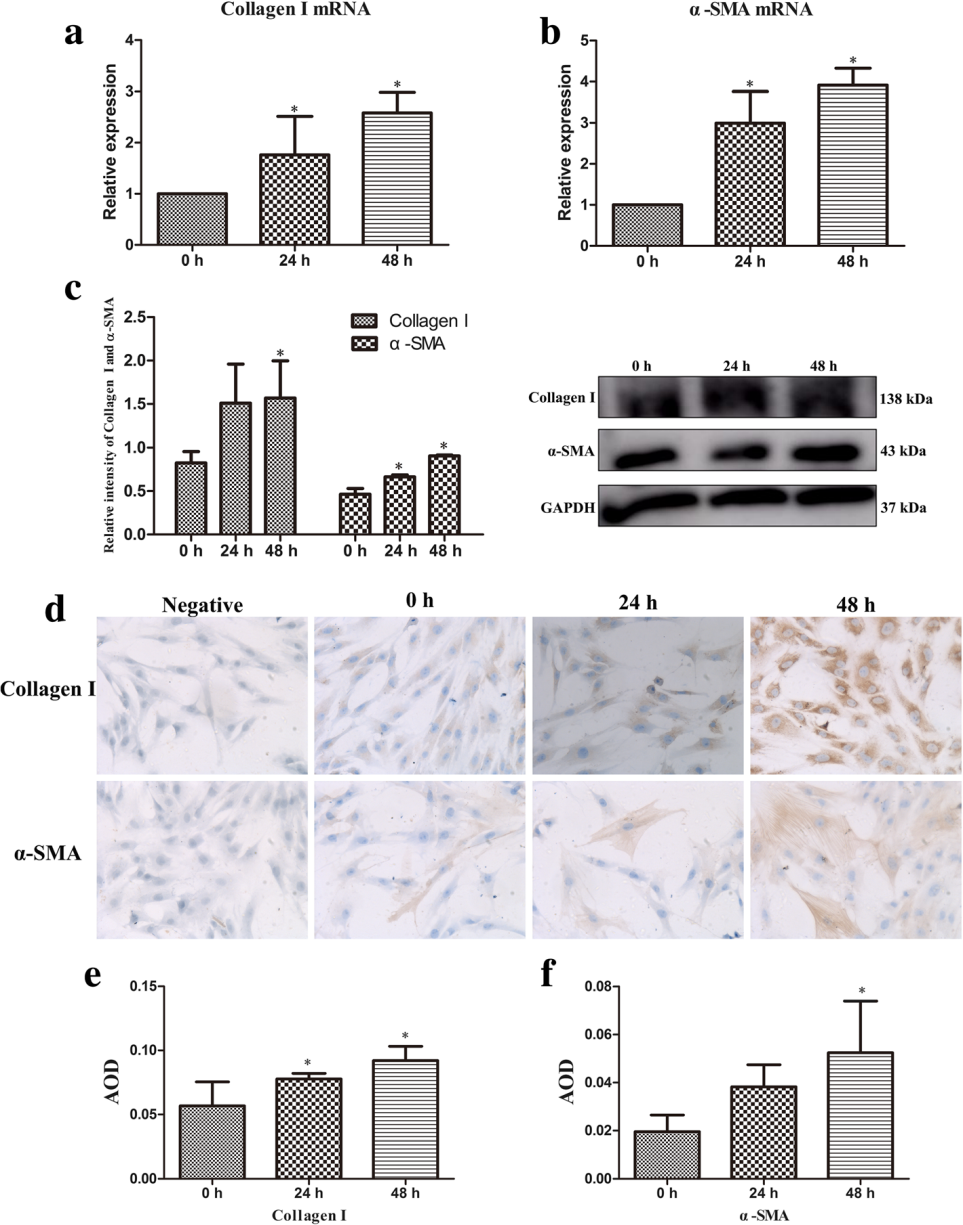
SiO_2_ exposure induces collagen I and α-SMA mRNA and protein expression in co-culture system in vitro. **a** and **b** Expression of collagen I and α-SMA mRNA in fibroblast in SiO_2_ exposure co-culture system, mRNA expression of SiO_2_ treated groups is compared to control group. **c** Levels of collagen I and α-SMA protein expression in fibroblast were examined by western blotting and normalized to those of GAPDH. **d**, **e** and **f** Collagen I and α-SMA protein expression were measured by IHC (magnification 200×). All values represent the mean ± SD in three separate experiments. *P < 0.05 compared with control group

mRNA expression of collagen I and α-SMA in fibroblast in co-culture system were investigated by qPCR. Collagen I and α-SMA mRNA in 24 h and 48 h treatment groups were significantly increased compared to those of 0 h treatment group (*P* ≤ 0.05; Fig. 1a and b). Expression of collagen I and *α*-SMA proteins was determined using western blotting and IHC. As shown in Fig. 1c, SiO_2_ exposure increased the expression of collagen I and *α*-SMA in fibroblasts both at 48 h treatment group (*P* ≤ 0.05). IHC data also revealed collagen I and *α*-SMA protein expression were higher than those of 0 h treatment group (*P* ≤ 0.05; Fig. 1d, e and f)

### MeDIP-seq data validation

In the present study, two regions were selected to carry out bisulfite sequencing to validate the MeDIP-seq data. Bisulfite sequencing results were consistent with the MeDIP-seq data, suggesting that our sequencing data were reliable (Additional file 6).

### Global DNA methylation changes in different treatment groups and further filtering of DMGs

**Fig. 2:**
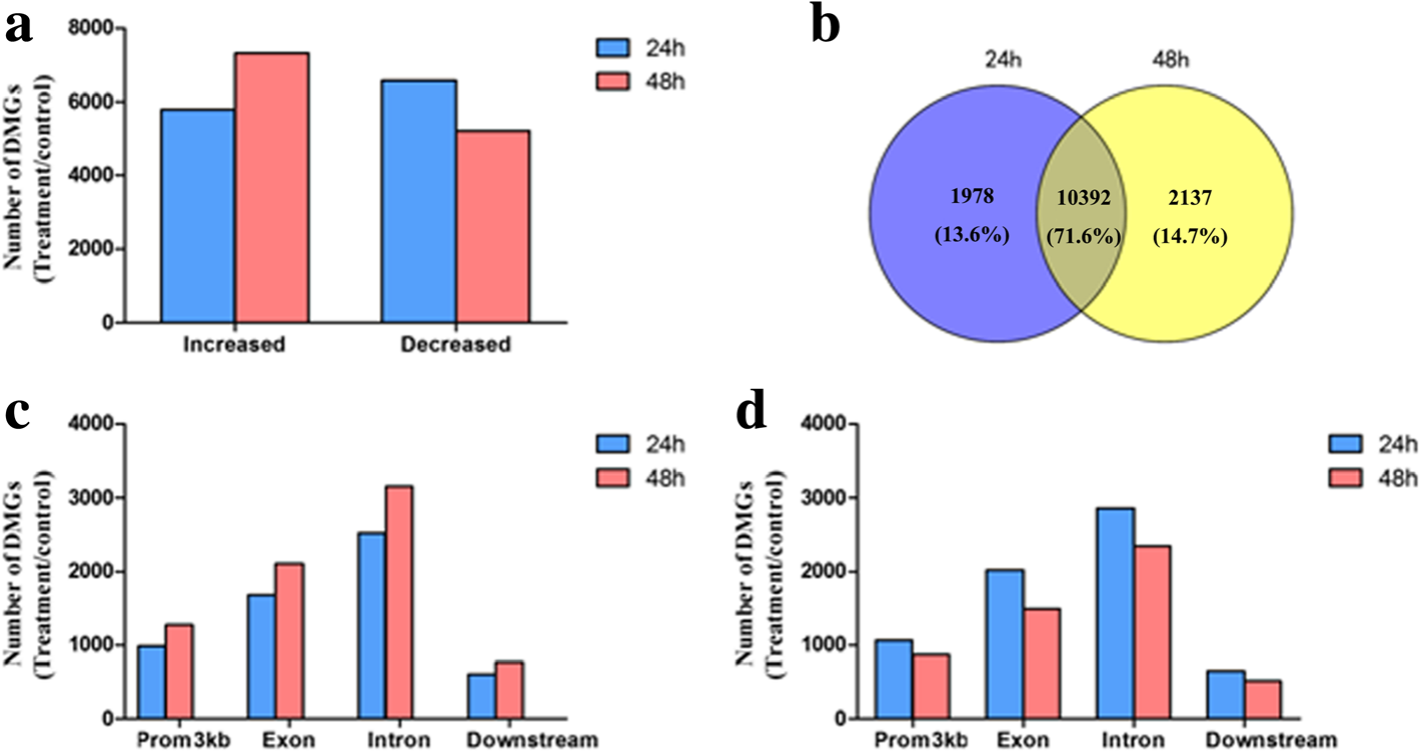
Number and distribution of DMGs in the 24- and 48-h groups. **a** The total number of significantly increased and decreased genes based on changes in methylation. **b** Composition of DMGs in the two treatment groups. **c** Distribution of hypermethylated genes in different genomic regions. **d** Distribution of hypomethylated genes in different genomic regions

As shown in Fig. 2a, in the 24 h treatment group, 12370 DMGs were identified in the samples that had a ≥2-fold change in peak reads. Compared with the control group, 5788 DMGs were increased, and 6582 genes were decreased. However, in the 48 h treatment group, 12529 genes were identified compared to the control group. Among these genes, the methylation pattern was increased in 7307 genes and decreased in 5222 genes. 10392 genes were identified with a ≥2-fold change in both of the two treatment groups (Fig. 2b). Figure 2c and d show the density distribution of these DMGs is over the whole genome The DMGs of the two groups were both enriched in exonic and intronic sequences. Compared with the 24 h group, the number of hypermethylated DMGs was increased and that of hypomethylated DMGs was decreased.

Through further filtering (Log_2_ Fold-change ≥2 or Log_2_ Fold-change ≤−2; *P*-value ≤0.05; 24 h exposure group vs. control and 48 h exposure group vs. control), we obtained 7246 (24 h group; 3529 hypermethylated and 3717 hypomethylated) and 5463 (48 h group; 2793 hypermethylated and 2770 hypomethylated) DMGs. Among these DMGs, the level of methylation of DMGs both increased (1015) and decreased (1827) in the two treatment groups.

### Functional and pathway analysis

**Fig. 3:**
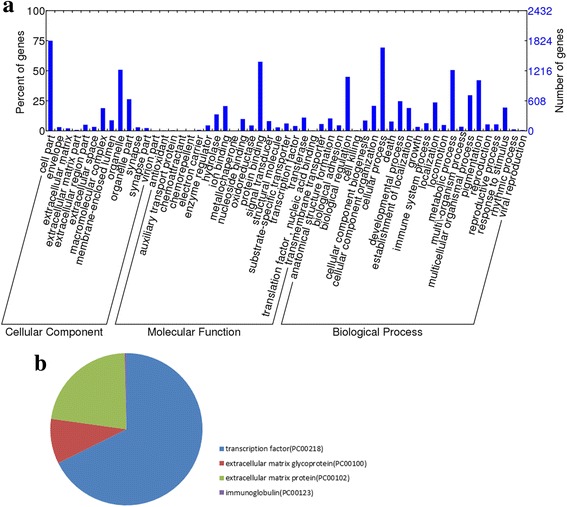
The significant GO categories and protein categories of DMGs. **a** The DMGs were clustered in three functional categories, including cellular component, molecular function and biological process. **b** PANTHER protein class categories clustered in “transcription factor”, “extracellular matrix glycoprotein” and “extracellular matrix protein”

We performed GO analysis and KEGG analysis to classify functions and the most prominent pathways of 2842 DMGs. Figure 3a shows the significant GO categories among these genes. In the cellular component category, there are several GO categories enriched in “extracellular matrix” and “extracellular space”. Molecular function showed enrichment of the terms “protein binding”, “ion binding” and “transferase”. The most abundant GO terms were enriched in “metabolic process” and “biological regulation” in the biological process category. In addition, a number of genes were also enriched in “biological adhesion”.

Through a KEGG pathway analysis, we found that the significantly hypermethylated and hypomethylated genes were involved in several pathways, including “Metabolic pathways”, “Regulation of the actin cytoskeleton” and “Focal adhesion”, while the hypomethylated genes were also enriched in the “MAPK signaling pathway”. In Table 2, the top five significant KEGG pathways among hypermethylated and hypomethylated DMGs were listed. The related genes of each pathway were also listed in Additional file 7. To further study the genes, the PANTHER website was used for protein classification. The DMGs were enriched in the categories “transcription factor” and “extracellular matrix protein” (Fig. 3b).

**Table 2:** KEGG pathway analysis of upregulated and downregulated DMGs determined using Web Gestalt

Rank	Name	Ratio	Observation/total	p-Value
hypermethylated genes				
1	Metabolic pathways	0.041	48/1169	2.22E-06
2	Pathways in cancer	0.053	17/319	0.0005
3	Regulation of actin cytoskeleton	0.072	15/208	6.96E-05
4	Focal adhesion	0.070	13/186	0.0004
5	Insulin signaling pathway	0.091	12/131	6.96E-05
hypomethylated genes				
1	Metabolic pathways	0.091	107/1169	4.29E-22
2	Pathways in cancer	0.084	27/319	7.84E-05
3	MAPK signaling pathway	0.085	23/269	0.0002
4	Endocytosis	0.095	22/230	7.84E-05
5	Focal adhesion	0.112	21/186	1.46E-05

**Fig. 4:**
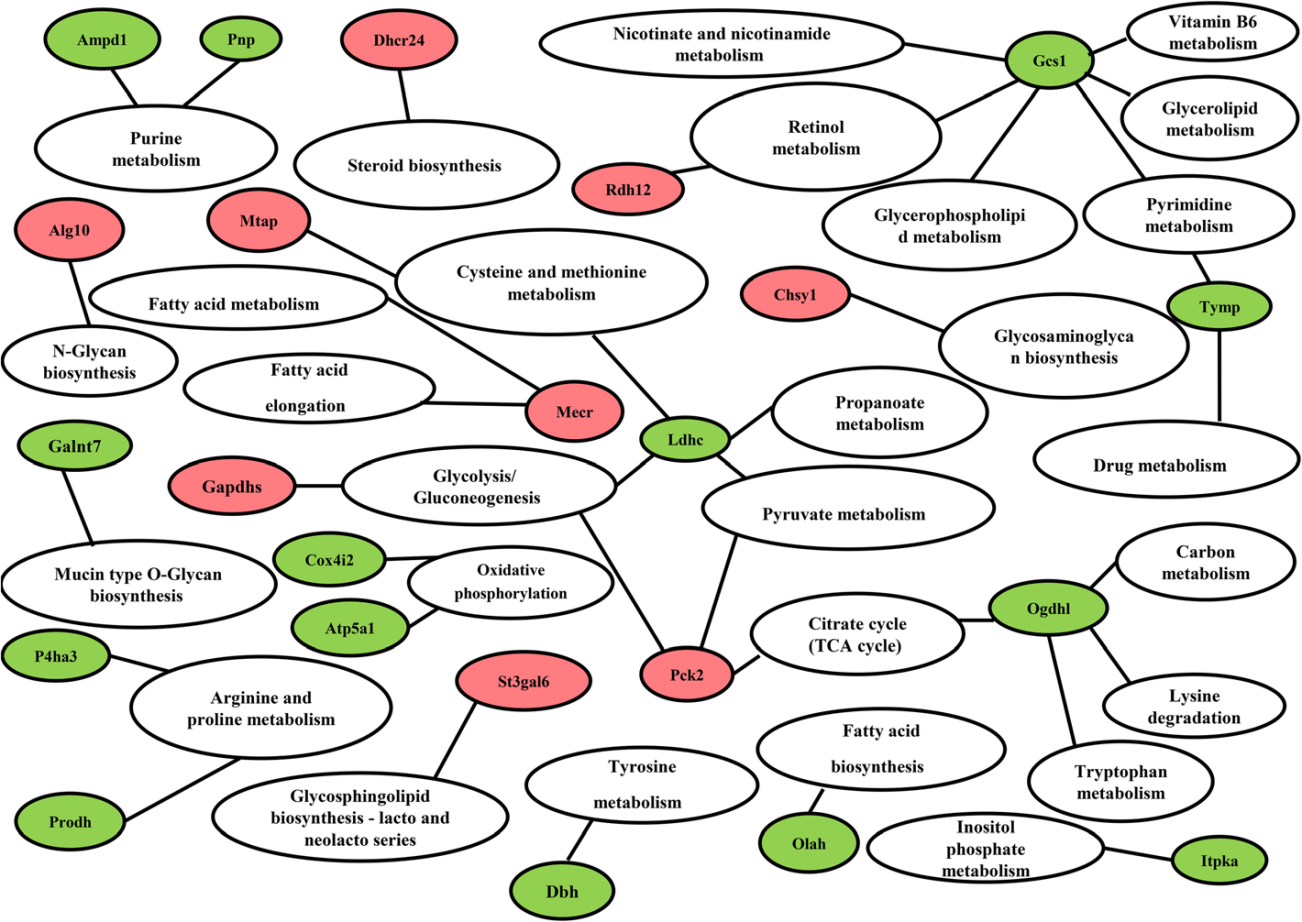
KEGG analysis of upregulated and downregulated promoter DMGs showing involvement in a metabolic pathway. (Red, hypermethylated genes; green, hypomethylated genes; white, KEGG pathways)

We further analyzed 25 promoter methylated genes enriched in “Metabolic pathways”. In Fig. 4, 23 genes were entered into the KEGG pathway analysis, which showed they were clustered in carbohydrate metabolism [including “Glycolysis/Gluconeogenesis”, “Citrate cycle (TCA cycle)”, “Pyruvate metabolism”, and “Propanoate metabolism”], energy metabolism [“Oxidative phosphorylation”] and amino acid metabolism [including “Cysteine and methionine metabolism”, “Tyrosine metabolism”, and “Arginine and proline metabolism”]. It is interesting that *Ldhc*, *Pck2*, *Gcs1* and *Ogdhl* participated in multiple metabolic pathways.

### Module screening from the PPI network

**Fig. 5:**
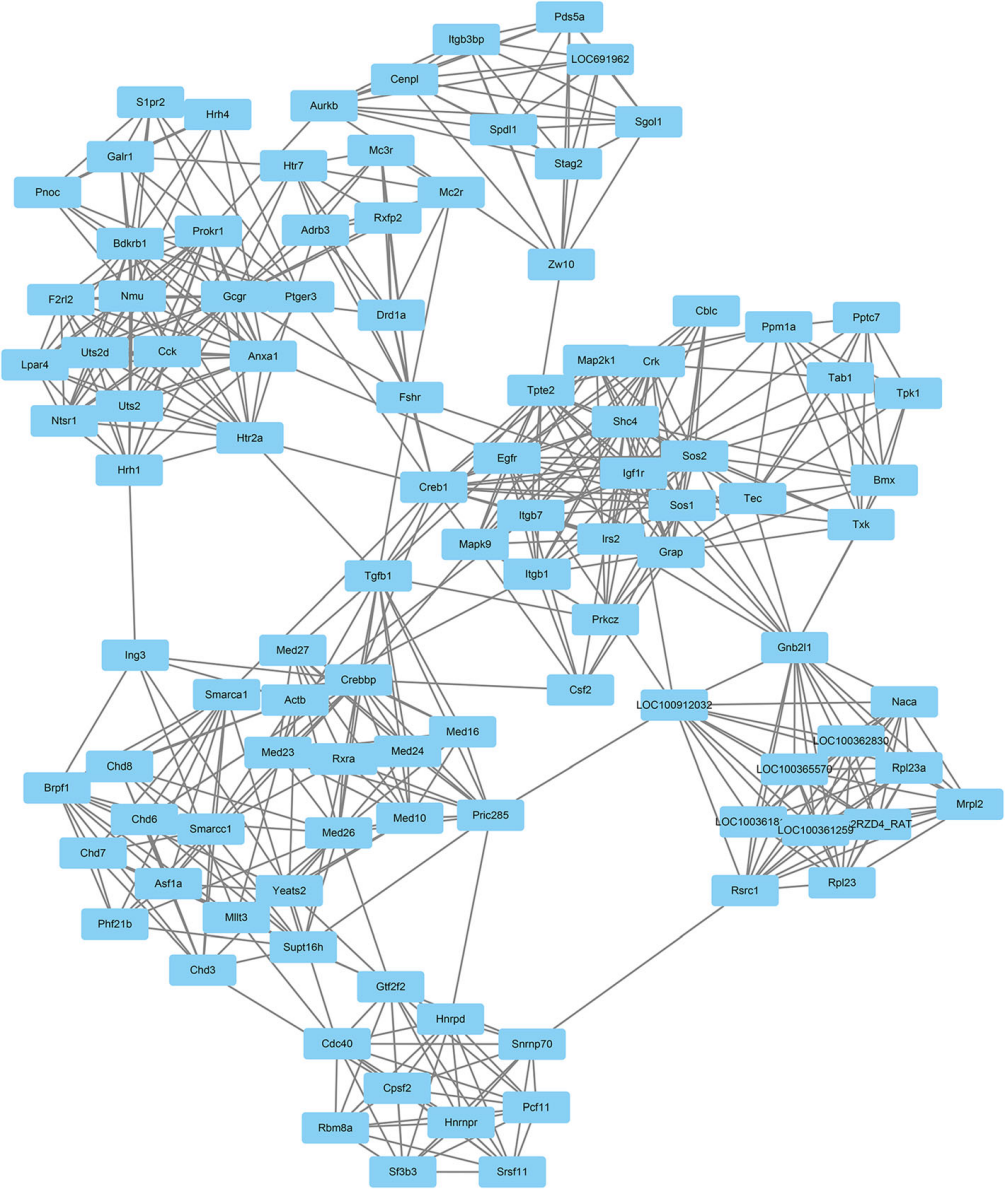
Module from the protein-protein interaction network

Using the Cytoscape 3.2.0 plugin String, a total of 2134 nodes and 10892 edges were analyzed using plug-ins MCODE. The top 2 significant modules were selected and the functional annotation of the genes involved in module 2 were analyzed (Fig. 5). Enrichment analysis showed that the genes in module 2 were mainly associated ErbB signaling pathway, Focal adhension and MAPK signaling pathway (Table 3). As shown in Fig. 5, *Tgfb1* is a hub who can connect three gene clusters. Moreover, *Mapk9* is also associated with all the three pathways. Therefore, further study of these genes should be performed to explore their molecular mechanisms in myofibroblast differentiation.

**Table 3:** Enriched pathways of module 2 from the protein-protein interaction network

Gene Set	P-value	FDR	Nodes
ErbB signaling pathway	3.50E-05	3.70E-02	Egfr, Map2k1, Sos1, Sos2, Mapk9, Crk, Shc4
Focal adhension	8.10E-05	8.60E-02	Egfr, Map2k1, Sos1, Sos2, Spp1r12a, Mapk9, Crk, Itgb1, Shc4
MAPK signaling pathway	1.20E-04	1.30E-01	Egfr, Map3k7ip1, Map2k1, Sos1, Sos2, Ppm1a, Mapk9, Cacna1c, Crk, Tgfb1

### Effect of SiO_2_ on TGF-β1 and MAPK9 mRNA and protein expression in fibroblasts

**Fig. 6:**
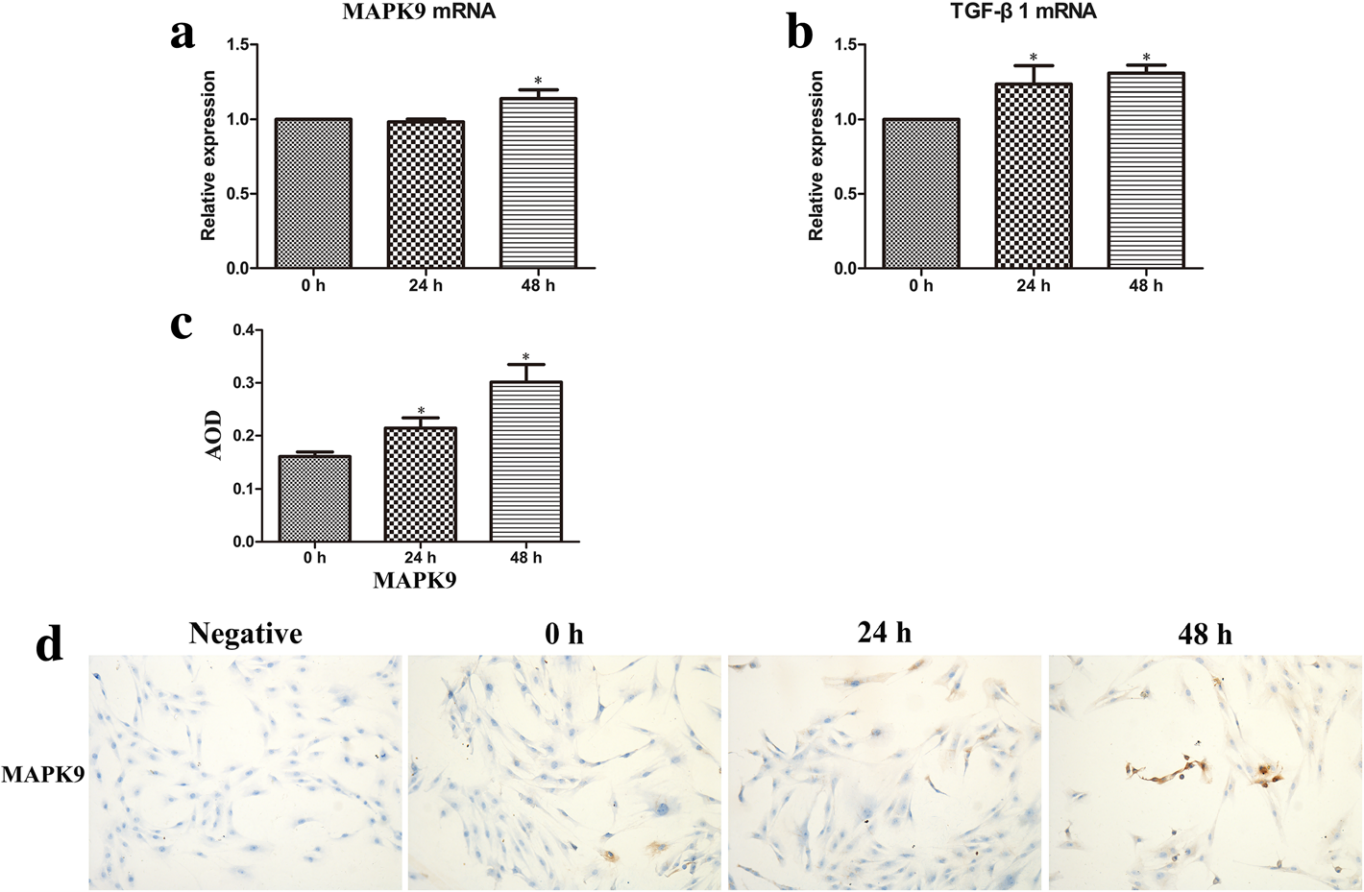
SiO_2_ exposure induces MAPK9 and TGF-β1 mRNA and protein expression in co-culture system in vitro. **a** and **b** MAPK9 and TGF-β1 mRNA expression in fibroblast in SiO_2_ exposure co-culture system, mRNA expression of SiO_2_ treated groups is compared to control group. **c** and **d** MAPK9 protein expression was measured by IHC (magnification 100×). All values represent the mean ± SD in three separate experiments. *P < 0.05 compared with control group

qPCR and IHC were used to identify the TGF-β1 and MAPK9 mRNA and protein expression. As shown in Fig. 6a and b, TGF-β1 and MAPK9 mRNA in fibroblasts were higher than those of 0 h treatment group after exposed to SiO_2_ for 48 h (*P* ≤ 0.05). The MAPK9 protein expression of SiO_2_ treatment groups was increased compared to that of 0 h treatment group (Fig. 6c and d). However, we didn’t find TGF-β1 protein expression in all the three groups (data not shown).

## Discussion

Until now, the underlying mechanisms involved in silica inhalation are still not clear, it is generally accepted that the AMs is a relevant cell to study [20]. Due to the capable of clearing the inhaled debris in lung, it is reasonable to assume that AM is the first cell of the body that will have significant contact with the inhaled silica particle. Myofibroblast is also known to be the key effector responsible for lung fibrosis [1]. Studies have demonstrated that crystalline silica can induce fibroblast switching into a myofibroblast [21, 22]. Several studies have also reported that DNA methylation may play an essential role in myofibroblast differentiation [12, 23–25]. However, few studies have reported fibroblast methylation profiling in vitro.

At present, extensive efforts are emphasized on the rebuilding in vitro of the complexity of the in vivo state so as to contribute to better reproduce the physiological and structural relationships in which cells are involved in vivo [26]. An exposure system such as co-culture model in vitro was widely used to identify the mechanisms by SiO_2_-induced adverse health effects [26–28]. Our studies provide the first comprehensive, detailed map of DNA methylation patterns in lung fibroblasts co-cultured with silica-exposed alveolar macrophages. The CCK8 result shown half maximal inhibitory concentration (IC50) of AMs exposed to SiO_2_ is 104.84 μg/ml, So we choose 100 μg/ml as our stimulating dose. We further found that fibroblast can easily differentiate into myofibroblast in co-culture system after SiO_2_ exposure. In our following study, we identified multiple DMGs, and these methylation differences spanned the genome. The methylation sites are not only in the promoters of genes but also distributed throughout the gene, including exons, introns and downstream regions. Greater attention is paid to methylation changes in the promoter; fewer studies focus on methylation in the other regions. However, we made an interesting observation that more DMRs are located within introns. Our study suggests that methylation sites in the other regions may participate in the biological process of myofibroblast differentiation.

In this study, we identified 2842 DMGs of which 427 altered regions are in the promoter. Gene ontology analysis revealed that certain DMGs were enriched in several annotations, such as “extracellular” and “extracellular space”. Protein enrichment analyses also showed differentially methylated genes were enriched in the annotation for extracellular matrix proteins. These GO terms are associated with a disorder that is characterized by tissue remodeling due to excess deposition of matrix proteins such as collagens. Majority types of collagen can be found in lung, 90% of which is collagen I and collagen III. Collagens are continually synthesized and degraded to keep a dynamic equilibrium in normal. Fibroblast is known to be the major producer of collagen in the lung [29]. Thus, excessive deposited of collagens found in fibrotic areas could be due to the fibroblast proliferation, or enhanced synthesis or fibroblast-like cell from other source recruited to the injured site [30]. Extensive research has reported that fibroblasts could give rise to a myofibroblast phenotype with high synthetic capacity for ECM proteins [31–33]. However, it is not known how fibroblasts differentiate into myofibroblast. In our study, we also observed that AMs exposed to SiO_2_ can promote fibroblast differentiation and enhance the secreting of collagen I. These results suggested that DNA methylation may participate in the fibroblast differentiation and further affect the synthesis of ECM. Further identifying these genes mapping to the GO terms may provide an insight into the myofibroblast differentiation mechanism.

Several interesting pathways with altered methylation/demethylation, such as “Focal adhesion”, “Regulation of the actin cytoskeleton” and the “MAPK signaling pathway”, were identified through a KEGG pathway analysis. Our study revealed that hypermethylated and hypomethylated genes were both enriched in “Focal adhesion”. Researchers have already reported that the category “Focal adhesion” is closely related to fibroblast activation or differentiation. Cell adhesion/integrin-FAK signaling can induce differentiation of myofibroblast by TGF-β1 [34, 35]. Conversely, proliferation and differentiation of cardiac fibroblasts into myofibroblasts can be inhibited by FAK knockdown. A number of hypermethylated genes were exclusively enriched in “Regulation of actin cytoskeleton”. The ECM, integrin-associated adhesions and the actin cytoskeleton are known to play a critical role in the physical interaction of a fibroblast with its extracellular environment [36]. The actin cytoskeleton is viewed as a hub for triggering the subsequent transcriptional events during myofibroblast differentiation. GTPases, such as Rho and rac, regulate the polymerization of actin to produce α-SMA stress fibers in the myofibroblast, which can also be activated by a number of profibrotic ligand-receptor complexes [37, 38]. A study of human atrial fibroblasts found that inhibition of RhoA geranylgeranylation may prevent the adverse myocardial remodeling associated with cardiac myofibroblast proliferation. In addition, downstream genes of Rho, such as ROCK1 and mDia, which are both required for polymerization and bundling stress fibers, also participate in myofibroblast differentiation. Knockdown of mDia may inhibit α-SMA transcription and myofibroblast differentiation in fibroblasts [39]. Cytoskeletal remodeling and matrix gene expression were also blocked by pharmacologic inhibition of ROCK1 in TGF-β-stimulated fibroblasts [40–42]. Moreover, ischemic hearts from ROCK1 knockout mice do not develop fibrosis and show a reduction in the number of myofibroblasts [43]. Non-canonical TGF-β signaling is closely related to myofibroblast differentiation [44]. This signaling is primarily involved in phosphorylating several intermediate MAPKs through activating the downstream TGF-β receptor kinase 1 (TAK1), which then activates p38 or c-Jun N-terminal kinase (JNK) signaling [36]. The most interesting observation from our study is that p38 (*Mapk14*) and c-Jun (*Mapk9*) genes are methylated, indicating that their methylation status may influence myofibroblast differentiation, which is regulated by MAPK signaling. It is remarkable that many other pathways explored were associated with myofibroblast differentiation and fibrotic diseases. The interaction between these pathways will ultimately determine the differentiated state of the fibroblast, and further research is needed to confirm this.

Metabolic disorder is associated with the pathogenesis of idiopathic pulmonary fibrosis (IPF). Our study found most DMRs clustered in “Metabolic pathways”, and further analysis of promoter DMRs showed they were mainly clustered in genes involved in “Glycolysis/Gluconeogenesis” such as *Ldhc*, *Pck2*, *Gapdhs* and *Pck2,* which displays promoter hypermethylation, is involved in blocking glucose metabolism. Promoter methylation is widely regarded as inhibitory to gene expression. The hypermethylated status of *Pck2* downregulated its gene expression and promoted glucose metabolism. Several studies have identified a connection between glycolysis and myofibroblast differentiation. Chen and colleagues found that quiescent hepatic stellate cells differentiate into myofibroblast depending on the induction of aerobic glycolysis [45]. Xie and colleagues also observed aerobic glycolysis can produce succinate, which may indirectly promote myofibroblast differentiation by TGF-β. They also noted that partially blocking glycolysis by inhibiting 6-phosphofructo-2-kinase/fructose-2, 6-biphosphatase 3 is effective in suppressing human lung fibroblast differentiation into myofibroblast [46]. A study by Bernard and colleagues also supports the view that myofibroblast contractility and differentiation are related to metabolic reprogramming, which is associated with the activation of the p38 mitogen-activated protein kinase (MAPK) pathway [47]. Although studies have found myofibroblast differentiation is linked to a dysregulation of cellular metabolism, the exact mechanism is unclear. In particular, how DNA methylation/demethylation participates in this process and whether they play a pivotal role is still unknown. More researches are needed to study the role of these dysregulated pathways of cellular metabolism in fibrosis diseases and take them together with available genetic and epigenetic data.

Module analysis of the PPI network further revealed that ErbB signaling pathway, Focal adhesion and MAPK signaling pathway were associated with myofibroblast differentiation in a co-culture model. *Tgfb1* and *Mapk9* are mainly involved in the MAPK signaling pathway. Abundant evidence implicates TGF-β1, which is mainly involved in cell signaling pathways such as the focal adhesion kinase pathway, JNK/p38 and PI3K/Akt pathways, as a key mediator of fibroblast activation and differentiation [35, 48]. Pan and colleagues found that TGF-β1 can downregulate the expression of DNMT1 and DNMT3 as well as inhibit the global DNMT activity, which may cause further DNA demethylation of the COL1A1 promoter and induce collagen type I expression in rat cardiac fibroblasts [49]. McDonnell and colleagues revealed that decreased promoter methylation of TGFb1 with increased expression of the TGFb1 gene in the GLC cells is correlated with fibrotic changes in glaucomatous eyes [50]. The activation of JNK can also stimulate cellular proliferation and transformation in HSC activation and fibrogenesis [51]. MAPK9, also known as JNK2, is a negative regulator of cellular proliferation in multiple cell types. The activity of JNK2 is required in the process of osteoblast differentiation [52]; however, there are few studies describing whether JNK2 participates in myofibroblast differentiation. Our module 2 showed that *Tgfb1* is a hub who connects three gene clusters, might be good candidates for further study of the process of lung myofibroblast differentiation. Moreover, *Mapk9* is also associated with all the three pathways. In the present study, MAPK9 and TGF-β1 mRNA were vertified to increase in the SiO_2_ treated sample. In addition, MAPK9 protein expression was also increased. These data indicates *Mapk9* and *Tgfb1* may particapte in the lung myofibroblast differentiation and changes of methylation status of these genes may be involved in this process. The exactly mechanism is still unknown and more work need to be done on this area.

## Conclusion

Our study shows DNA methylation and demethylation participate in lung myofibroblast differentiation caused by exposed to crystalline silica. The present study not only provides a profiling of the whole-genome DNA methylation patterns of lung fibroblasts but also presents several signaling pathways and genes involved in myofibroblast differentiation. Further studies will reveal the underlying mechanism for DNA methylation alteration in myofibroblast differentiation. Potential limitations of our present study are that effect on gene expression is not measured which limits functional relevance of the findings, the limited samples and limited scope of the study applicable to rats; however, these results can provide us some clues to study on epigenetic gene regulation in fibroblast differentiation in future study.

## Additional files

Additional file 1: Figure S1. The fibroblast/AM co-culture system in vitro. (TIF 213 kb)

Additional file 2: Table S1. The total number of reads generated by MeDIP-Seq for each sample. (DOCX 16 kb)

Additional file 3: Table S2. The total number of peaks. (DOCX 15 kb)

Additional file 4: Table S3. The primers and cycling condition for PCR. (DOC 47 kb)

Additional file 5: Figure S2. The cytotoxicity of AMs exposed to SiO2 for 24- and 48 h. (TIF 347 kb)

Additional file 6: Table S4. The result of bisulfite modified sequence analysis. (DOCX 16 kb)

Additional file 7: Table S5. KEGG pathway analysis of upregulated and downregulated DMGs. (DOCX 19 kb)

## Abbreviations

AM: Macrophages
AOD: Average optical density
BAL: Bronchoalveolar lavage
DAB: 3,3′-diaminobenzidine
DMEM: Eagle’s medium Dulbecco
DMGs: Differential methylation associate genes
DMRs: Differential methylation regions
ECM: Extracellular matrix
GAPDH: Glyceraldehyde-3-phosphate dehydrogenase
IC50: Half maximal inhibitory concentration
IHC: Immunohistochemistry
IOD: Integrated Optical Density
IPF: Idiopathic pulmonary fibrosis
KEGG: Kyoto Encyclopedia of Genes and Genomes
MACS: Model-based Analysis of MeDIP-Seq software
MCODE: Molecular Complex Detection
MeDIP: Methylated DNA Immunoprecipitation
SD: Sprague–Dawley
TGF-β1: Transforming growth factor-β
