# Supplementary figures and images for: Genome-wide DNA methylation analysis in lung fibroblasts co-cultured with silica-exposed alveolar macrophages
Source: Respir Res. 2017 May 12;18:91. doi: 10.1186/s12931-017-0576-z (PMC5429546; doi:10.1186/s12931-017-0576-z)

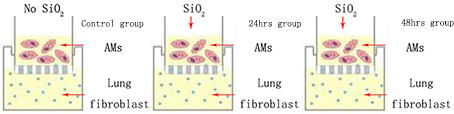

Supplement: Supplementary file 1 — The fibroblast/AM co-culture system in vitro. (TIF 213 kb) [file 12931_2017_576_MOESM1_ESM.tif]

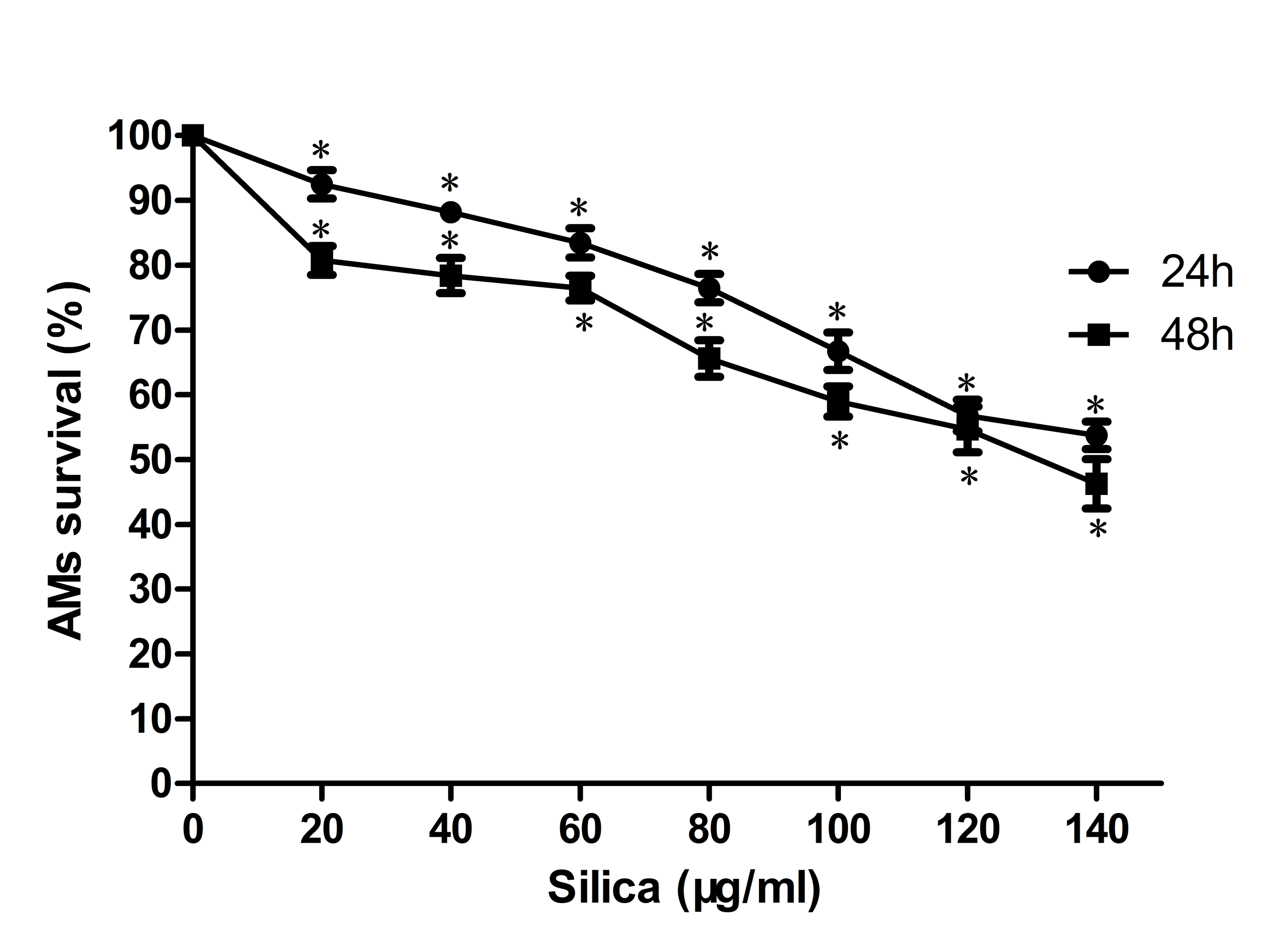

Supplement: Supplementary file 5 — The cytotoxicity of AMs exposed to SiO2 for 24- and 48 h. (TIF 347 kb) [file 12931_2017_576_MOESM5_ESM.tif]
