# Supplementary material for: Genome-wide DNA methylation analysis in lung fibroblasts co-cultured with silica-exposed alveolar macrophages
Source: Respir Res. 2017 May 12;18:91. doi: 10.1186/s12931-017-0576-z (PMC5429546; doi:10.1186/s12931-017-0576-z)
Supplement: Supplementary file 2 — The total number of reads generated by MeDIP-Seq for each sample. (DOCX 16 kb) [file 12931_2017_576_MOESM2_ESM.docx]

**S1 Table Number of reads generated by MeDIP-Seq for each sample**

|  | A1(0h) | A2(24h) | A3(48h) |
| --- | --- | --- | --- |
| Total number of reads | 26048428 | 34162578 | 33314252 |
| Total number of mapped read | 22192364 | 28930222 | 28256146 |
| Mapping rate (%) | 85.20 | 84.68 | 84.82 |
