# Supplementary material for: Genome-wide DNA methylation analysis in lung fibroblasts co-cultured with silica-exposed alveolar macrophages
Source: Respir Res. 2017 May 12;18:91. doi: 10.1186/s12931-017-0576-z (PMC5429546; doi:10.1186/s12931-017-0576-z)
Supplement: Supplementary file 3 — The total number of peaks. (DOCX 15 kb) [file 12931_2017_576_MOESM3_ESM.docx]

**S2 Table The total number of peaks**

|  | A1(0h) | A2(24h) | A3(48h) |
| --- | --- | --- | --- |
| Number of peaks | 208253 | 206685 | 232504 |
| Total length of peaks | 151108854 | 164141300 | 178122709 |
| Mean length of peaks | 725.6 | 794.16 | 766.11 |
| Median length of peaks | 645 | 692 | 678 |
