# Supplementary material for: Genome-wide DNA methylation analysis in lung fibroblasts co-cultured with silica-exposed alveolar macrophages
Source: Respir Res. 2017 May 12;18:91. doi: 10.1186/s12931-017-0576-z (PMC5429546; doi:10.1186/s12931-017-0576-z)
Supplement: Supplementary file 4 — The primers and cycling condition for PCR. (DOC 47 kb) [file 12931_2017_576_MOESM4_ESM.doc]

S3 Table **PCR primers and cycling condition**

| chromosome | location | Forward primer | Reverse primer | Cycling Conditions  98°C 4min  94°C 45sec  20×  66°C 45sec  72°C 1min  94°C 45sec  20×  56°C 45sec  72°C 1min  72°C 8min |
| --- | --- | --- | --- | --- |
| 1 | 101625428-101626541 | TTAGGTTTTAGAGTTTAGGTAGTAGTTG | AAAATCTCTCCCTATTTATCTTTCTC |
| 18 | 150210027-150211027 | GTTAAATAGAAGAATGTAGTTGTTGTTAG | AAAATAAACTCATCCCCAATACAC |
|  |  |  |  |
|  |  |  |  |
|  |  |  |  |
