# Supplementary material for: Genome-wide DNA methylation analysis in lung fibroblasts co-cultured with silica-exposed alveolar macrophages
Source: Respir Res. 2017 May 12;18:91. doi: 10.1186/s12931-017-0576-z (PMC5429546; doi:10.1186/s12931-017-0576-z)
Supplement: Supplementary file 6 — The result of bisulfite modified sequence analysis. (DOCX 16 kb) [file 12931_2017_576_MOESM6_ESM.docx]

**S4 Table The result of Bisulfite modified sequence analysis**

| Location | TPM value | | | BSP methylation rate (%) | | |
| --- | --- | --- | --- | --- | --- | --- |
|  | A1 | A2 | A3 | a1 | a2 | a3 |
| Chromosome1 101625428-10162654 | 0 | 4.379 | 3.191 | 1.75 | 2.75 | 3.00 |
| Chromosome18 150210027-150211027 | 0 | 5.474 | 3.003 | 36.25 | 73.75 | 48.75 |
