# Supplementary material for: Genome-wide DNA methylation analysis in lung fibroblasts co-cultured with silica-exposed alveolar macrophages
Source: Respir Res. 2017 May 12;18:91. doi: 10.1186/s12931-017-0576-z (PMC5429546; doi:10.1186/s12931-017-0576-z)
Supplement: Supplementary file 7 — KEGG pathway analysis of upregulated and downregulated DMGs. (DOCX 19 kb) [file 12931_2017_576_MOESM7_ESM.docx]

**S5 Table KEGG pathway analysis of upregulated and downregulated DMGs.**

| Rank | Name | Ratio | Observation/total | EntrezGene | p-Value |
| --- | --- | --- | --- | --- | --- |
| **hypermethylated genes** | | | |  |  |
| 1 | Metabolic pathways | 0.041 | 48/1169 | Crls1,Abat,Slc27a5,Me3,Pon1,  Gldc,Gapdhs,Pgap1,Aldh7a,  Lipt1,Dao,St3gal6,Mecr,Xylt1,  Mboat1,Fut8,Anpep,Gfpt2,  Pik3c2b,Rdh12,Gda,Pla2g5,  Mthfd2,Ido2,Lpcat4,Rdh10,  Cyp4f6,Lipg,Mtap,Dhcr24,  Umps,Rev3l,Pole2,Synj2,  Impa2,Dnmt1,Pck2, Chsy1  LOC690550,Ldha,Pah,Lias,  Lpcat1,Dpys,Alg9,Galnt1,  Alg10, | 2.22E-06 |
| 2 | Pathways in cancer | 0.053 | 17/319 | Pik3r1,Max,Fgf12,Birc3,Ikbkb,  Fzd6,Tp53,Apc,Tpm3,Pax8,Crk,Sos1,Flt3,Igf1,Ctnna1,Faslg,  Arn2 | 0.0005 |
| 3 | Regulation of actin cytoskeleton | 0.072 | 15/208 | Arhgef7,Pik3r1,Itgb8,Itgb6,  Apc,Sos1,Fgf12,Actg1,Itgb7,  Gsn,Limk2,Limk1,Wasl,Itga11,Crk | 6.96E-05 |
| 4 | Focal adhesion | 0.070 | 13/186 | Parvb,Pik3r1,Itgb8,Itgb6,  Actg1,Birc3,Itgb7,Src,Igf1,  Itga11,Crk,Capn2, Sos1 | 0.0004 |
| 5 | Insulin signaling pathway | 0.091 | 12/131 | Gys2,Pik3r1,Prkcz,Phkb,Ikbkb,  Flot2,Prkci,Pck2,Socs2,Crk,  Sos1,Pygb | 6.96E-05 |
| **hypomethylated genes** | | | |  |  |
| 1 | Metabolic pathways | 0.091 | 107/1169 | Csgalnact1,Polr3c,Alox15,  Olah,Pccb,B4galt2,Tph2,  Suclg1,Lss,Cat,Galnt7,Chpt1,  Ogdhl,Atp5a1,Agxt,Dhfr,Ada,  Ugt2a3,Ndst1,Pkm,Nadsyn1,  Pnp,Mpst,Mboat2,Shmt2,  B4galt1,Atp6v1a,H6pd,  Pon2,Dhrs3,Shmt1,Aldh1a3,  Sord,Nadk,Tbxas1,Sptlc2,Pgk2,  Gss,Synj1,Hmgcl,Acadm,  Inpp4a,Coq3,Sardh,Galns,  Sgpl1,Mgat4a,Ccbl1,Atp6v0e1,Pnliprp2,Dguok,Gcs1,Gne,Hgd,Inpp5a,St3gal4,Cox4i2,Gls,  Acadsb,Pi4k2b,Hao1,Asah1,Dhrs9,Ces1e,Gfpt1,Rpia,Cyp2c22,Msmo1,Gmds,Ampd1,Ldhc,  Hal,Sqle,Dck,Ugdh,Aox3l1,  Mthfd1,Alad,Pla2g4a,Ndufs3,  Dbh,Acer2,Tdo2,Itpka,RGD1310572,Got2,Itpkb,Hmgcs1,Coq6,Papss1,Cds2,Pla2g6,Tymp,  Ugt8,Xdh,Aldh2,Uqcrc2,P4ha3,Dgat1,Ndufa5,Prodh,Hibadh,  Gmppb,Ggps1,Smpd3,Hsd17b3 | 4.29E-22 |
| 2 | Pathways in cancer | 0.084 | 27/319 | Dcc,Ptch1,Cblc,Vegfc,Hras,  Hsp90ab1,Tgfb1,Mecom,Rxra,  Mmp9,Map2k1,Pdgfrb,Casp9,  Fgf6,Mapk9,Vegfa,Pml,Bcl2l1,  Runx1,Itgb1,Gsk3b,Egfr,Ccne1,  Col4a2,Egln1,Wnt8b,Sos2 | 7.84E-05 |
| 3 | [MAPK signaling pathway](http://www.kegg.jp/pathway/rno04010+309168+500526+24629+81737+315139+24666+29748+293621+170700+25387+24239+294924+59086+170851+140729+65189+24653+24329+360426+50658+81649+85384+170920) | 0.085 | 23/269 | Pla2g6,Map3k11,Ppp3r1,  Mknk1,Hras,Tgfb1,Mecom,  Map2k1,Pdgfrb,Fgf6,Cacng8,  Mapk9,Ntf3,Stk3,Pla2g4a,Tab1,Mapk14,Egfr,Arrb1,Cacna1c,  Sos2,Map43,Ppm1a | 0.0002 |
| 4 | Endocytosis | 0.095 | 22/230 | Adrb3,Rab11b,Fam125b, Cav3,  RT1-M6-1,Cblc,RT1-CE7, Tgfb1,Smurf2,Hras,Pml,Ap2a1,Dnm2,Ehd4,Ap2m1,Dnm1l,  Wwp1,Egfr,Rab7a,Agap3,  Arrb1,Cav1 | 7.84E-05 |
| 5 | Focal adhesion | 0.112 | 21/186 | Itga5,Cav3,Parvg,Vegfc,Hras,  Pdgfd,Tln1,Pdgfc,Map2k1,  Pdgfrb,Ppp1r12a,Sos2,Mapk9,  Vegfa,Itgb1,Gsk3b,Egfr,Col4a2,  Cav1,Col5a1,Col11a2 | 1.46E-05 |
